# Supplementary material for: Toward Pair Atomic Density Fitting for Correlation Energies with Benchmark Accuracy
Source: J Chem Theory Comput. 2023 Feb 14;19(5):1499–516. doi: 10.1021/acs.jctc.2c01201 (PMC10018742; doi:10.1021/acs.jctc.2c01201)
Supplement: Supplementary file 1 — ct2c01201_si_001.pdf [file ct2c01201_si_001.pdf]

Supporting information to: Toward Pair Atomic Density Fitting for Correlation Energies with Benchmark Accuracy

Edoardo Spadetto,[*,†] Pier Herman Theodoor Philipsen,[*,†] Arno Förster,[*,†,‡] and Lucas Visscher[‡]

†*Software for Chemistry and Materials NV, NL, 1081HV, Amsterdam, The Netherlands*

‡*Theoretical Chemistry, Vrije Universiteit, De Boelelaan 1083, NL-1081 HV, Amsterdam, The Netherlands*

E-mail: spadetto@scm.com; philipsen@scm.com; a.t.l.foerster@vu.nl

S1 Settings

Settings needed to reproduce our results are shown in S1 through BAND input files. The used development version includes new keywords which are expected to be kept in the new release version. The keyword `Method FromBasisProducts` switches on the fit set algorithm described in the main text. `OneCenterDependencyThreshold` is the ϵ_{fit} threshold, and finally `boostL` enables the l-e method. To enable Löwdin orthonormalization we use the `dependency` block with keyword `allowbasisdependency` which, if not present, leads to a stop of the calculation if unsafe linear dependencies are present in the basis set. In the same block it is possible to specify ϵ_{bas} through the `bas` keyword. In ADF, the same new keywords have been introduced as can be seen in table S2.

Table S1: BAND settings when compared with Psi4, `boostL` that is l-e is set to true only for `CC-TZPGTO`. `CC-XZPGTO` are exactly the Dunnings correlation consistent basis sets, cc-PVXZ. The Slater basis `TZ2PSTO` is equal to `TZ2P` in ADF. `OneCenterDependencyThreshold` i.e. ϵ_{fit} is always set equal to 10^{-12} except for some large molecules which for computational reasons it is set to 10^{-10}. These molecules are listed in table S3. The keyword `bas` by default is set to 10^{-8}

```
Engine BAND
  Basis
    Core None
    Type CC-TZPGTO ( or CC-QZPGTO, CC-5ZPGTO, TZ2PSTO )
  End
  MBPT
    dependency TRUE
  End
  NumericalQuality VeryGood
  RIHartreeFock
    DependencyCoreRange 0.0
    DependencyThreshold 1e-3
    FitGenerationDetails
      Method FromBasisProducts
      OneCenterDependencyThreshold 1e-12 (1e-10)
      boostL True (or False)
    End
    FitSetQuality VeryGood
  End
  Relativity
    Level None
  End
  SoftConfinement
    Quality Excellent
  End
  XC
    HartreeFock
    MP2
  End
  dependency
    AllowBasisDependency
    (bas 1e-4)
  End
EndEngine
```

Table S2: Settings of ADF compared with BAND

```
Engine ADF
  Basis
    Core None
    Type TZ2P
  End
  MBPT
    dependency True
  End
  NumericalQuality VeryGood
  RIHartreeFock
    DependencyCoreRange 0.0
    DependencyThreshold 1e-3
    FitGenerationDetails
      OneCenterDependencyThreshold 1e-12
    End
    FitSetQuality FromBasisProducts
  End
  XC
    MP2
  End
  symmetry nosym
EndEngine
```

S2 Reduced fit quality for 5ζ

In table S3 we list molecules for which 5ζ simulation needed a lower fit quality. This was necessary to overcome the memory large consumption during generation of the fit functions.

Table S3: Molecules for which cc-PV5Z fit set had quality $\epsilon_{\text{fit}} = 10^{-10}$ instead of 10^{-12}

Index	Molecule
43	UracilNeopentane
35	NeopentanePentane
37	CyclopentaneNeopentane
35	NeopentanePentane
41	UracilPentane
34	PentanePentane
37	CyclopentaneNeopentane

S3 Absolute correlation energies for dimers in the L7 set

Table S4: Convergence of absolute SOS-MP2 correlation energies using the CC-pVTZ basis set for the 7 dimers in the L7 dataset with respect to the size of the fit set, controlled by the value of the threshold N_{fit}. All values are in Hartree. We have used $\epsilon_K = 5 \times 10^{-3}$.

System	1e-6	1e-8	1e-10
c2c2PD	−7.559 143	−7.557 230	−7.556 402
c3a	−10.153 443	−10.151 534	−10.150 137
c3gc	−11.903 990	−11.901 741	−11.900 096
cbh	−6.766 746	−6.764 397	−6.763 496
gcgc	−7.010 118	−7.009 139	−7.008 690
ggg	−6.098 328	−6.097 669	−6.097 413
phe	−8.209 136	−8.207 467	−8.206 631

S4 Basis set convergence for CIM8 Set

We here list the non-covalent interaction energies of the molecules in the Cim8 for different basis sets and different extrapolation schemes, calculated with and without counterpoise corrections.

Table S5: Interaction energies calculated with different basis sets and extrapolation schemes for the first molecule in the CIM8 set, "Capsule" with and without counterpoise corrections. Δ denotes the difference between the corrected and the non-corrected results. All values are in kcal/mol.

cp correction [%]	DZ	TZ	QZ	(D,T)	(T,Q)
100	−39.86	−52.51	−60.17	−58.22	−64.86
0	−70.50	−66.08	−53.44	−67.80	−56.93
Δ	30.64	13.58	−6.73	9.58	−7.93

Table S6: Interaction energies calculated with different basis sets and the corresponding extrapolated results for all molecules in the CIM8 set with and without counterpoise corrections. All values are in kcal/mol.

	100 % cp			0 % cp		
System	DZ	TZ	(D,T)	DZ	TZ	(D,T)
1	−39.86	−52.51	−58.22	−70.50	−66.08	−67.80
2	−30.26	−47.76	−55.76	−75.49	−62.15	−62.79
3	−23.46	−28.29	−31.01	−40.74	−34.46	−34.67
4	−8.91	−12.67	−14.09	−15.10	−20.91	−24.90
5	−18.10	−29.40	−34.31	−39.93	−43.65	−48.67
6	−21.39	−47.42	−58.97	−86.19	−79.77	−86.81
7	−254.48	−306.17	−336.83	−390.89	−391.67	−414.75
8	−6.47	−19.94	−25.58	−29.43	−35.45	−41.27

S5 Computational Timings

In this section we briefly comment on the timings and asymptotic scaling of our algorithm with respect to basis set and fit set size. For a detailed analysis of the asymptotic scaling of our algorithm to calculate the non-interacting polarizability we refer to our earlier work.[1,2] There, we have also shown that the evaluation of the non-interacting polarizability (which scales quadratic with system size) is typically the compute-time determining step.

In figure S1 and figure S2 we show the scaling of SOS-MP2 calculations with respect to the size of the single-particle basis and the size of the fit set, using the PADF-based and the canonical algorithm. We use here the example of the GGG complex from the L7 set with 48 atoms in total.

Since this molecule is rather small no major speed-ups of the AO-based algorithm compared to the canonical one can be expected due to sparsity in the single-particle basis. In figure S1, the fit set is generated from basis products and therefore its size increases proportionally with the size of the single-particle basis. Therefore, since the canonical DF-SOS-MP2 algorithms scales as $N_{occ}^2 N_{virt}^2 N_{fit}$, the compute time will increase as the cube of the single-particle basis. The same can be expected for the AO-based algorithm when sparsity in the AO basis is not exploited.[1]

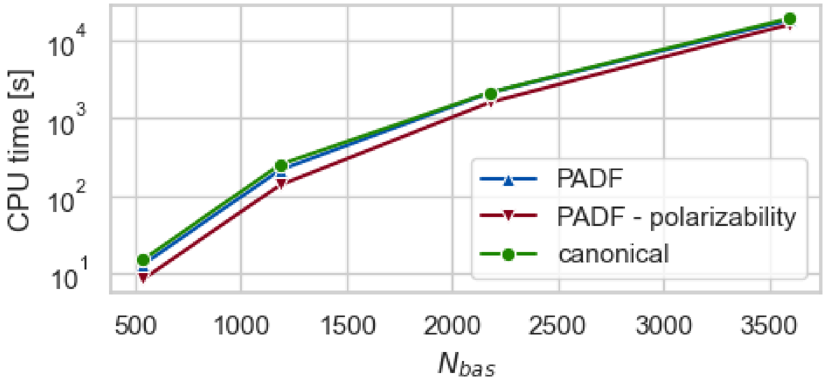

Figure S1: Computational timings of PADF-SOS-MP2 and DF-SOS-MP2 algorithms using different basis sets ranging from cc-pVDZ to cc-pV5Z. The timings are in seconds. Calculations have been performed on a single AMD compute node (zen 3 architecture) with 32 cores and 256 GB of RAM.

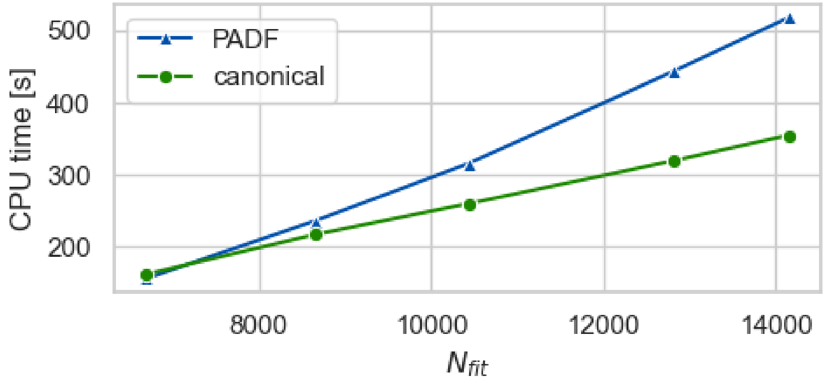

Figure S2: Computational timings of PADF-SOS-MP2 and DF-SOS-MP2 algorithms using different fit sets. The timings are in seconds. Calculations have been performed on a single AMD compute node (zen 3 architecture) with 64 cores and 256 GB of RAM.

Overall, the computational timings of both algorithms are comparable for different sizes of the single particle basis, ranging from cc-pVDZ to cc-pV5Z. However, the canonical algorithm shows better scaling with respect to the size of the fit set. It should be noted, that the size of the auxiliary basis in typical canonical, global DF based implementations is typically about a factor of two smaller than in our implementation. Therefore, our canonical algorithm is most likely about a factor of two slower than optimized DF-MP2 algorithms.

References

(1) Förster, A.; Franchini, M.; van Lenthe, E.; Visscher, L. A Quadratic Pair Atomic Resolution of the Identity Based SOS-AO-MP2 Algorithm Using Slater Type Orbitals. *J. Chem. Theory Comput.* **2020**, *16*, 875 – 891.

(2) Förster, A.; Visscher, L. Low-Order Scaling G0W0 by Pair Atomic Density Fitting. *J. Chem. Theory Comput.* **2020**, *16*, 7381–7399.
